# Combination of Stem Cell Mobilized by Granulocyte-Colony Stimulating Factor and Human Umbilical Cord Matrix Stem Cell: Therapy of Traumatic Brain Injury in Rats 

**Published:** 2011

**Authors:** Mehrdad Bakhtiary, Mohsen Marzban, Mehdi Mehdizadeh, Mohammad Taghi Joghataei, Samideh Khoei, Mahdi Tondar, Vahid Pirhajati Mahabadi, Bahareh Laribi, Asghar Ebrahimi, Seyed Jafar Hashemian, Navid Modiry, Soraya Mehrabi

**Affiliations:** 1 *Cellular & Molecular Research Center, Tehran University of Medical Sciences, Tehran, Iran*; 2 *Department of Anatomy, Tehran University of Medical Science, Tehran, Iran*; 3 *Department of Biotechnology and Bioscience, Milano-Bicocca University, Milan, Italy*; 4 *Department of Medical Physics, Tehran University of Medical Sciences, Tehran, Iran*; 5 *Department of Immunology, Tehran University of Medical Sciences, Tehran, Iran*; 6 *Department of physiology, Tehran University of Medical Sciences, Tehran, Iran*

**Keywords:** Combine Therapy, G-CSF, Stem Cell, TBI, Wharton, s jelly

## Abstract

**Objective(s):**

Clinical studies of treating traumatic brain injury (TBI) with autologous adult stem cells led us to examine the impression of a combination therapy. This was performed by intravenous injection of human umbilical cord matrix stem cell (hUCMSC-Wharton^,^s jelly stem cell) with bone marrow cell mobilized by granulocytecolony stimulating factor (G-CSF) in rats injured with cortical compact device.

**Materials and Methods:**

Adult male Wistar rats (n= 50) were injured with controlled cortical impact device and divided into five groups. All injections were performed 1 day after injury into the tail veins of rats. Neurological functional evaluation of animals was performed before and after injury using modified neurological severity scores (mNSS). Animals were sacrificed 42 days after TBI and brain sections were stained by Brdu immunohistochemistry.

**Results:**

Statistically significant improvement in functional outcome was observed in treatment groups when compared with control (*P*< 0.01). mNSS showed no significant differences among the hUCMSC and G-CSF treated groups at any time point (end of trial). Rats with hUCMSC + G-CSF treatment had a significant improvement on mNSS at 5 and 6 week compared to other treatment group (*P*< 0.01).

**Conclusion:**

Histological analysis in G-CSF+ hUCMSC treated traumatic rats exhibited significant increase in numbers of Brdu immunoreactive cells in their traumatic core compared with other labeled group.

## Introduction

Restorative strategy for traumatic brain injury (TBI) and neural injury has taken needful two ways, cellular and pharmacological. The cellular approach inclusive bone marrow stromal cells (MSC) (1-6) and pharmacological idea comprises medication with neurotrophic and growth factors (7-10) and other compositions, like granulocytecolony stimulating factor (G-CSF) (11,12). What has not been proved in study of restorative therapeutic insights to the medication of traumatic brain injury is whether pharmacological and cell therapies can be applied in combination and whether they interact to produce augmentation of the single therapeutic insight. Hence, in this project we examined the opinion that injection of human umbilical cord matrix stem cell (hUCMSC) and G-CSF when applied in combination to treat brain trauma enhances the therapeutic effect of the single therapy. The reason for combining therapies is that both the hUCMSC and G-CSF have the same and supplementary effect on the damaged brain. HUCMSC look like to BMSC (bone marrow stromal stem cells) enhance endogenous plasticity, angiogenesis and neurogenesis (13-15). Functional effect was from these cells, caused not from their capability to differentiate into cerebral tissue and functionate as stem or progenitor cells, but possibly from their power to enhance endogenous restorative process, by excitation the secretion of neurotrophic factors, for example VEGF and bFGF (13). It proved that G-CSF causes mobilization of hematopoietic stem cells (HSCs) from bone marrow into peripheral blood (16). Peripheral blood-derived HSCs have been applied for the regeneration of nonhematopoietic tissues for example skeletal muscle and heart (17). G-CSF has been applied for 10 years in the medication of neutropenia, also for bone marrow restructuring and stem cell mobilization (6). Wharton’s jelly (WJ) from the umbilical cord is a new region of stem cells (18). Particular immunohistochemical and ultra structural studies have proved that the cells from WJ of umbilical cords have specification of stromal cells. In last-mentioned investigations, matrix cells from umbilical cord WJ have been proved to differentiate into neural-like cells both *in vitro *and *in vivo* (19) and (20). In this project we have cultured a cell population extracted from the WJ of human umbilical cords which was developed *in vitro* and maintained in culture. We applied these cells to study migration and efficacy of intravenous injection of hUCMSC in traumatic brain injury in rats. 

## Materials and Methods

***Animal model***

**Figure 1:**
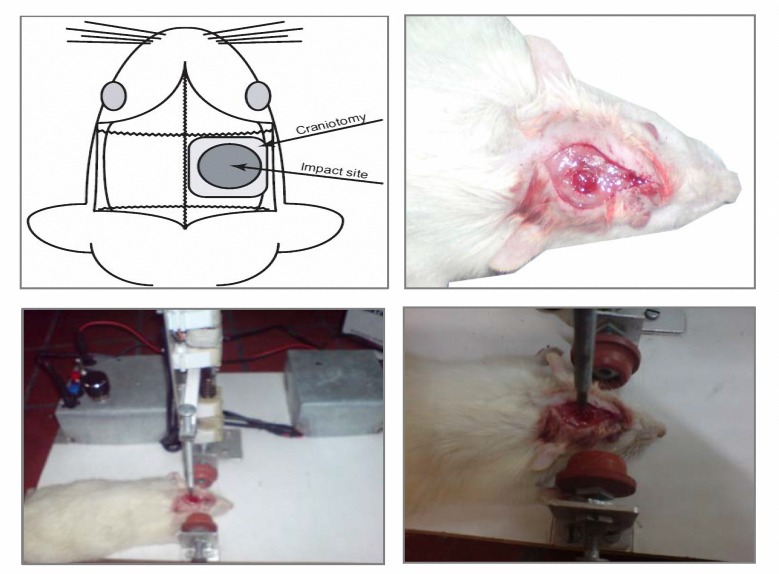
The (controlled cortical impact) set-up consists of an piston causing a mechanical trauma in mice and results in cortical contusions, morphological and cerebrovascular effects, subdural and intraparenchymal hematoma, edema, inflammation and changes in cerebral blood flow which clearly mimics human traumatic brain injury.

Adult male Wistar rat’s weights 250 to 300 g were used in this study. Animals were held in a room at 23 °C±2, humidity 45% to 55% with a fixed 12-h artificial light period and allowed to eat and drink *ad libitium*. All animals were fed with standard rodent diet and received human care, as outlined in the guide for the care and use of laboratory animals. This study was approved by Ethical Committee of Iran University of Medical Sciences () base on Principles of Laboratory Animal Care (NIH publication #85−23, revised in 1985). A controlled cortical impact device (Figure 1) was used to induce the injury. Rats were placed in device and 6-mm diameter craniotomies (Figure 1) were performed adjacent to the central suture, midway between lambda and bregma. The dura was kept intact over the cortex. Injury was induced by impacting the right cortex (ipsilateral cortex) with a piston containing a 5-mm diameter tip at the rate of 4 m/s and 2.5 mm of contusion (21).

Animals were divided into five groups as follows:

Group 1 (10): TBI +PBS

Group 2 (10): TBI + Brdu (intraperitoneally)

Group 3 (10): TBI +G-CSF (subcutaneous) +Brdu (intraperitoneally)

Group 4 (10): + 2 × 10^6^ hUCMSC

Group 5 (10): TBI +2 × 10^6^ hUCMSC+G-CSF + Brdu (intraperitoneally)

***Culture of human umbilical cord matrix stem cell***

The collected human umbilical cord tissues were washed three times with Ca^2+^ and Mg^2+^-free PBS. They were mechanically cut by scissors in a midline direction and the vessels of the umbilical artery, vein and outlining membrane were dissociated from the WJ. The jelly was then extensively cut into pieces smaller than 0.5 cm^3^, treated with collagenase type 1 (Sigma) and incubated for 14-18 hr at 37 °C in a 95% air/5% CO_2_ humidified atmosphere. The explants then were cultured in DMEM containing 10% fetal calf serum (FCS) and antibiotics at 37 °C in a 95% air/5% CO_2_ humidified atmosphere. They were left undisturbed for 5-7 days to allow for migration of the cells from the explants. The cellular morphology became homogenously spindle-shaped in cultures after 2-3 passages (19). This cell also labeled with 8-9 μl of DiI ((170 mg/ml in DMSO and diluted 1:10 in saline.

***Alkaline phosphatase (AP) staining procedure***

We used CHEMICON’s Alkaline Phosphatase Detection Kit (Catalog number SCR004). HUCMSC (hUCMSC) cells were cultured for five days prior to analyzing AP activity at low to medium density. According to our findings, five days after culturing spindle-shaped cells are optimal for good AP stain visualization. On day five, media were aspirated and hUCMSC were fixed with a fixative (4% paraformaldehyde in PBS) for 1-2 min. Fixative were aspirated and rinsed with 1 X rinse buffer (TBST: 20 mM Tris-HCl, pH 7.4, 0.15 NaCl, 0.05% Tween-20). Reagents for alkaline phosphatase staining naphthol/fast red violet solution: mix fast red violet (FRV) with naphthol AS-BI phosphate solution and water in a 2:1:1 ratio (FRV: naphthol: water) was prepared. Enough stain solution was added to cover each well (e.g. 0.5 ml for a well of a 24-well plate) and then incubated in dark at room temperature for 15 min. Staining solution was aspirated and wells were rinsed with 1 X rinse buffer. The cells were covered with 1 X PBS to prevent drying and then the numbers of colonies were counted.

***Differentiation protocols***

To confirm the differentiation potential of hUCMSC, P5 cells were plated at 5000 cells/cm^2^ in four well chamber slides and re-incubated in α-MEM for 10 days. The differentiative protocols for adipogenesis were used as it shown in below.

***Adipogenesis***

Cells were incubated in α-MEM that was accompanied with 20% FCS, 100 U/ml penicillin, 100 mg/ml streptomycin, 12 mM L-glutamine, 5 mg/ml insulin (sigma), 50 mM indomethacin (Sigma), 1 mM dexamethasone (Sigma) and 0.5 mM 3-isobutyl-1-methylxanthine (IBMX) for 2 weeks. Cells were ﬁxed with 10% formalin for 20 min at room temperature and stained with 0.5% Oil Red O () in methanol (Sigma) for 20 min at room temperature.

***Smooth muscle actin (SMA) immunocytochemistry***

HUCMSCs were cultured onto glass coverslips and authorized to reproduce for upto 2 weeks. Growth medium was replaced and the cells were fixed in 4% paraformaldehyde for 5 min continuation by leakage with 0.2% Triton-X-100 and blocked with a proper element (3% normal goat or donkey serum + 1% bovine serum albumin in PBS). Mouse monoclonal antibodies to SMA (Chemicon) diluted 1:100 and used for 1 hr at room temperature or overnight at 4 °C. Secondary antibodies (goat anti-mouse IgG-FITC conjugated, diluted 1:200 in PBS (Sigma)) were used for 1 hr at room temperature. Imaged on Olympus IX70 Fluorescent microscope were captured with Olysa software and processed for publication using CorelDrawor Photoshop with identical settings for control and experimental slides.

***Stem cell labeling and transplantation***

To identify cells derived from hUCMSC, 3 mg/ml Brdu (Sigma), a thymidine analog and marker of newly synthesized DNA, was added to the medium at 72 hr before transplantation. Upon harvesting, cells were isolated by treatment with 0.25% trypsin and 0.5 mM ethylenediaminetetraacetic acid (EDTA) at 37 °C for 10 min at room temperature. The digestion was stopped by adding FCS. Cells were then washed five times with PBS. HUCMSC were counted using a cytometer to ensure adequate cell number for transplantation. For immunostaining, hUCMSC were subcultured in chambered slides and more than 90% of hUCMSC were Brdu reactive. Approximately 2×10^6 ^hr UCMSC in 200 ml PBS (n= 10) or control fluid (200 ml PBS; n= 10) was slowly injected over a 5 min period into each rat tail vein. Immunosuppressant was not used in any animal in this study.

***In vivo bromodeoxyuridine labeling***

Brdu that is incorporated into the DNA of dividing cells during S-phase was used for mitotic labeling. The labeling protocol has been described previously by Zhang *et al* (22). Experimental rats (including 10 G-CSF-treated rats and 10 combine therapy rats) were injected intraperitoneally with Brdu (50 mg/kg) every 24 hr for 5 consecutive days. Rats were killed at 42 days traumatic brain injury.

***Experimental animals and G-CSF treatment***

One day after induction of traumatic brain injury, rats were injected subcutaneously with recombinant human G-CSF (50 µg/kg) per day; once daily for 5 days (23). Control animals were subjected to traumatic brain injury and injected with saline.

***Neurological functional evaluation***

Neurological function in the rats was assessed using the modified neurological severity scores (mNSS). The mNSS is composed of motor (muscle status, abnormal movement), sensory (visual, tactile and proprioceptive), reflex, and beam walking tests. In the severity scores of injury, one point is awarded for the inability to correctly perform the tasks or for the lack of a tested reflex. The higher the mNSS score is, the more severe the injury. The evaluation of all rats was started after TBI and performed after TBI at 1 week, and weekly thereafter. All measurements were performed by observers blinded to individual treatment (24) (Table 1).

***Brdu immunohistochemical assessment***

The brains of experimental rats were fixed by transcardial perfusion with saline, followed by perfusion and immersion in 4% paraformaldehyde. The cerebral injured tissues (5 mm) were cut into coronal paraffin blocks. A series of 6-μm-thick sections at various levels (100-µm interval) were cut from this block and were analyzed by fluorescent microscopy for Brdu immunostaining. DNA was first denatured by incubating each section in 50% formamide of 2X standard saline citrate at 65 °C for 2 hr, then in 2N HCl at 37 °C for 30 min, and finally rinsed in 0.1 mol/l boric acid with pH 8.5. Sections were then rinsed with Tris buffer and treated with 1% H_2_O_2_ to block endogenous peroxidase. After deparaffinization, tissues slide were incubated with the appropriate monoclonal antibody against Brdu in mouse (diluted at 1:100 in PBS) (sigma), 1 day. After they were washed with Tris buffered saline containing 0.1% Tween-20, the specimens were sequentially incubated for 10 to 30 min with secondary antibody (goat anti-mouse IgG-Rhodamine conjugated (dilution 1:60 in PBS (Chemicon)). Quantification of Brdu-immunoreactive cells was performed on paraffin-embedded tissue sections and was counted digitally with the use of a 40 objective lens via a computer imaging analysis system. Cerebral cells with uniform nuclear Brdu immunostaining were counted as previously described by Kuhn *et al* (25). All Brdu-reactive cells, with Brdu were counted throughout all 10 coronal sections. Images on Olympus IX70 fluorescent microscop were captured with Olysa software and processed for publication using CorelDrawor Photoshop with identical settings for control and experimental slides.

***Statistical analysis***

Statistical analysis was performed using one-way analysis of variance (ANOVA) followed by the post-hoc test using SPSS 15.0. The data are presented as the mean±SD (standard deviation). *P*< 0.05 was considered to denote statistically significant differences.

**Table 1:** A set of modified neurologic severity scores used to grade neurologic function (24).

**Modified neurological severity score (mNSS)**	
**Motor tests**	**Points**
**Raising the rat by the tail**	3
1 Flexion of forelimb	
1 Flexion of hindlimb	
1 Head moved more than 10^0^ to the vertical axis within 30 s	
**Walking on the floor (normal=0; mazimum=3)**	3
0 Normal walk	
1 inability to walk straight	
2 Circling toward the paretic side	
3 Falling down to the paretic side	
**Sensory tests**	2
1 placing test (visual and tactile test)	
2 proproceptive test (deep sensation, pushing the paw against the table edge to stimulate limb muscles)	
**Boam balance testes (normal=0; maximum=6)**	6
0 Balance with steady posture	
1 Grasps side of beam	
2 Hugs the beam and one limb falls down from the beam	
3 Hugs the beam and two limbs fall down from the beam, or spins on beam (>60 s)	
4 Attempts to balance on the beam but falls off (>40 s)	
5 Attemps to balance on the beam but falls off (>20 s)	
6 Falls off: no attempt to balance or hang on to the beam (<20 s)	
**Reflexes absent and abnormal movements**	4
1 Pinna reflex (a head shake when the auditory meatus is touched)	
1 Corneal reflex (an eye blink when the cornea is lightly touched with cotton)	
1 Startle reflex (a motor response to a brief noise from snapping a clipboard and paper)	
1 Seizures, myoclonus, myodystony	
**Maximum points**	18

One point is awarded for the inability to perform the task or for the lack of a tested reflex: 13-18-sever injury: 7-12-moderate injury: 1-6-mild injury

## Results

***Isolation and characterization of hUCMSC***

**Figure 2.**
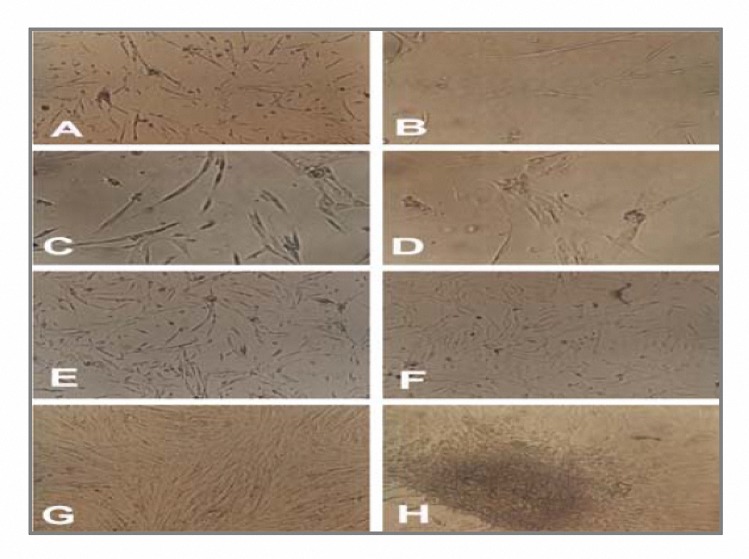
Pluripotential cells from the human umbilical cord matrix. (A-H) Appearance and growth of fibroblastic cells or human umbilical cord matrix stem cell at passage 0 on days 3, 7 (A-F), 12 (G) 14 (H) respectively. Briefly when hUCMSC cells initially grow outward from explants two morphologically distinct populations of cells are present: spherical or flat mesenchymal cells. When the cells become confluent, they form spherical colonies that remain attached to cells. These colonies resemble “neurosphere”.

Isolation, expansion and characterization of hUCMSC from human after preparing the WJ primary cultures, adherently growing cells of spindle shaped morphology were seen to migrate from the explants (Figure 2). Cells from WJ were fed and changed with culture medium twice weekly, and passaged as necessary. Freshly isolated cells (P_0_) principally displayed a fibroblast-like appearance over the first 3-4 days of culture (Figure 2A). During the 2nd week, they typically appeared as slender cells with narrow cytoplasm and few lamellopodia (Figure 2B-F)

**Figure 3.**
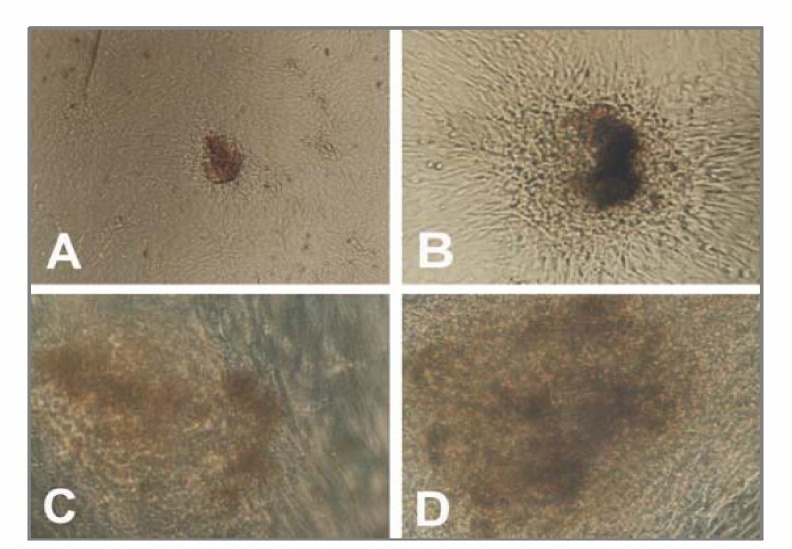
Human umbilical cord matrix stem cell colonies express alkaline phosphatase activity (A-D).

After 12-14 days, they grew to 100% confluence (Figure 2G) and formed colonies (Figure 2H) and P_1_ cells resembled P_0_ cells. We used alkaline phosphatase to detect stem cells and observed colonies express alkaline phosphatase (red or brown stem cell colonies) (Figure 3). HUCMSC cell culture can be maintained either by harvesting the neurosphere like cell clusters or by passage of preconfluent flat and spherical cells without apparent differences. We have maintained hUCMSC cultures for more than 50 population doublings and they continue to grow vigorously.

***Adipose differentiation and SMA immunocytochemistry***

**Figure 4.**
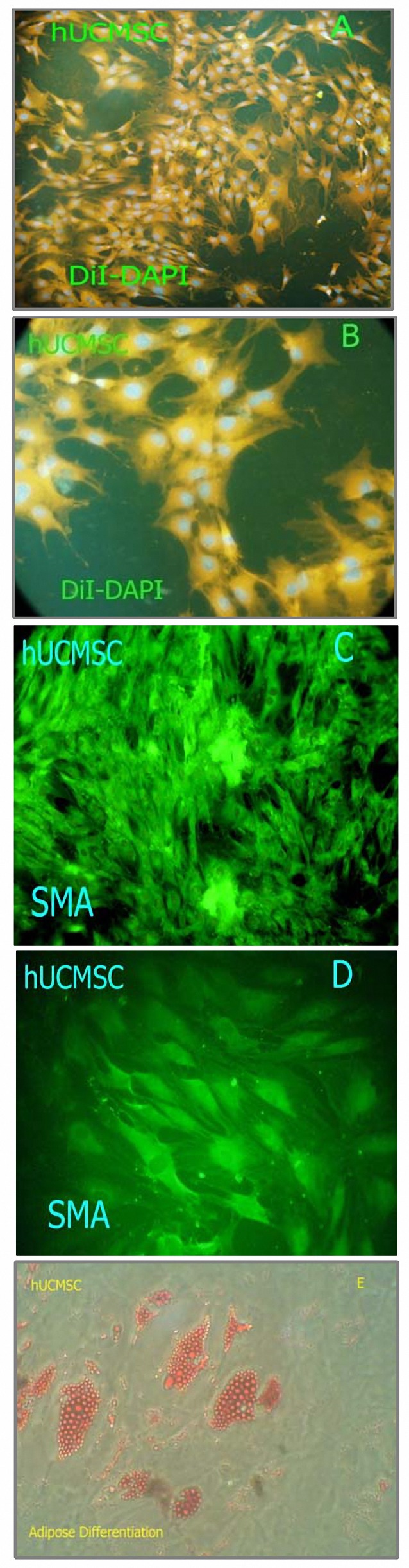
Characterization of human umbilical cord matrix cells (hUCMSC). Cells were stained by DiI and DAPI (A-B) or immunostained for smooth muscle actin (SMA) (C-D) (green). Differentiation potential of adherent cells isolated from hUCMSC. (E) Adipogenesis was detected by the formation of intra-cytoplasmic lipid droplets stained with Oil Red O.

Immunocytochemical examination was accomplished to test for expression of SMA protein. The cytoplasmic localization of the SMA filaments (green) is shown in (Figure 4D, C). Differentiation of hUCMSC into adipocytic lineages was observed. After adipogenic induction, the cell morphology changed from the elongated conﬂuent ﬁbroblastic cells to more oval shaped cells, which showed a distinct ring of red coarse vacuoles around the cell periphery after Oil Red O staining. These vacuoles appeared to develop by day 2 and became more numerous and larger with time (Figure 4E).

***Neurological and motor function evaluation***

**Figure 5.**
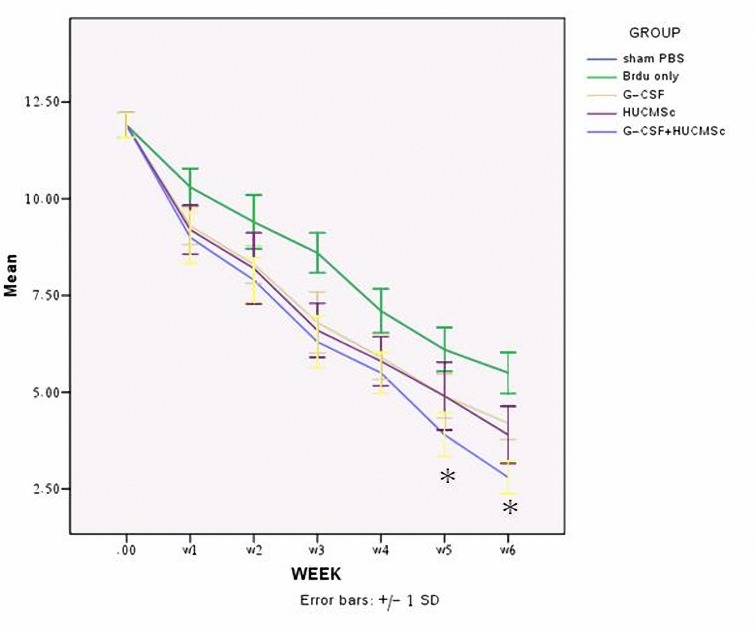
Results of behavioral functional tests (modified neurologic severity score [mNSS] test) before and after traumatic brain injury (TBI). Rats were injured to traumatic brain injury alone (Control) (n= 10) or were injected with cultured human umbilical cord matrix stem cell (hUCMSC) (n= 10) or Sham (PBS) (n= 10) or combine 1 day after TBI. Significant functional recovery was detected in rats treated with hUCMSC, G-CSF and hUCMSC+G-CSF compared with control and sham (*P*< 0.01). The data are presented as the Mean±SD=Week

Injury in the left hemispheric cortex of rats caused neurological functional deficits, as measured by the modified neurological severity scores (mNSS). These rats presented with high scores on motor, sensory, reflex, and beam balance tests in the early phase after injury (first week post injury). Recovery began in the end of first week and persisted at all subsequent evaluation times in all groups. Motor function tested by the mNSS recovered faster than sensory and beam balance functions. Rats with hUCMSc+G-CSF treatment had a more significant improvement on (mNSS) in 5 and 6 weeks compared to other treatment group (*P*< 0.01). In the functional outcome on mNSS in 6^th^ week between the G-CSF and hUCMSC treated group was not significant (Figure 5) (Table 2).

***Presence of Brdu positive cells***

**Figure 6.**
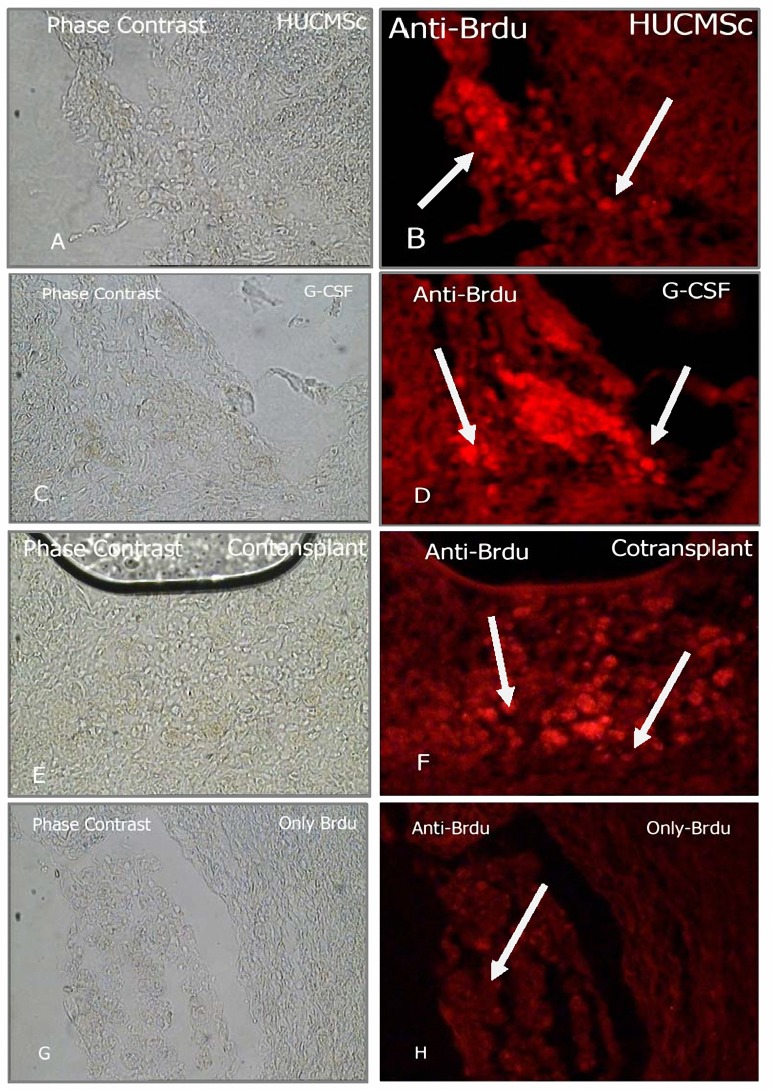
Presence of Brdu positive cell. Brdu immunoreactive cells 41 days after hUCMSC intravenous transplantation and G-CSF injection were identified by Rhodamine conjugated secondary antibody (B-red spot (HUCMSc), D-red spot (G-CSF mobilized Cell ) distribute in the territory of the TBI (40X) (Olympus IX70).

**Figure 7.**
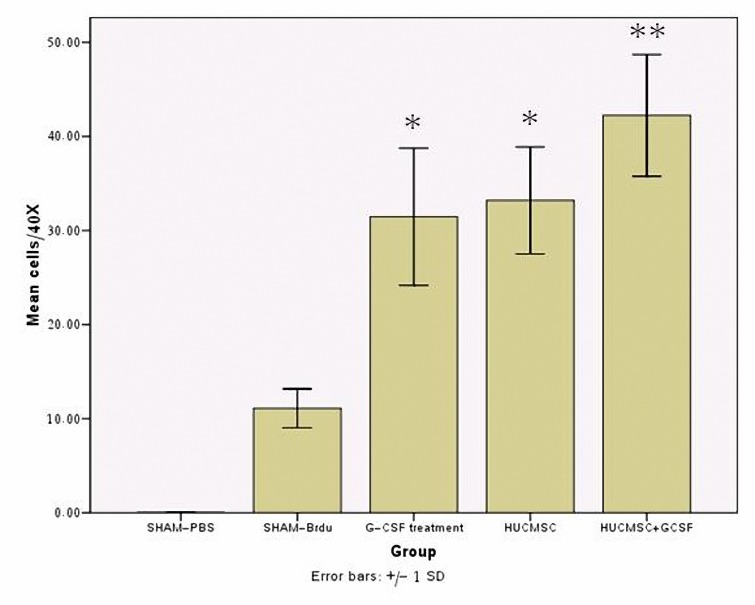
The G-CSF, hUCMSC, hUCMSC+G-CSF treated group showed a significant increase of BrdU-positive cells compared to the vehicle-Brdu treated group in the traumatic area at 42 days after TBI *P< 0.01. Each column and bar denotes mean±SD.

Brdu-reactive cells detected from an average of 10 histology slides per treatment animal multiple areas of the ipsilateral hemisphere, including cortexes, striatum of the ipsilateral hemisphere. Histological analysis showed that 6 week after administration, hUCMSC and G-CSF mobilized cells were still visible in the recipient brain, primarily around the lesion site, the vast majority of Brdu-labeled cells were located in the traumatic core and its boundary zone. Few cells were observed in the contralateral hemisphere. In summary G-CSF+hUCMSC treated traumatic rats exhibited significantly increased numbers of Brdu immunoreactive cells in their traumatic core compared with other labeled group. (Figures 6, 7) (Table 3).

**Table 2.** Statistical analysis was performed using one-way analysis of variance (ANOVA) followed by Duncan post-hoc test with SPSS 15.0 for modified neurological severity scores (mNSS).The data are presented as the mean±SD. Rats with hUCMSC+G-CSF treatment had a more significant improvement on (mNSS) in 5 and 6 weeks compared to other treatment group (*P*< 0.01).

Week	W1	W2	W3	W4	W5	W6
Group						
TBI+PBS	10.3±0.483	9.4±0.699	8.6±0.516	7.1±0.567	6.1±0.567	5.5±0.527
TBI+Brdu	10.3±0.483	9.5±0.707	8.6±0.516	7.1±0.567	6.1±0.567	5.5±0.527
TBI+G-CSF	9.3±0.483	8.3±0.483	6.8±0.788	5.9±0.567	4.9±0.567	4.2±0.421
TBI+HUCMSc	9.2±0.632	8.2±0.918	6.6±0.699	5.7±0.674	4.7±0.948	3.9±0.737
TBI+HUCMSc+G-CSF	9±0.66	7.9±0.576	6.3±0.674	5.5±0.527	3.9±0.567	2.8±0.421

**Table 3.** The count of BrdU cells (Means±SD). In the G-CSF + hUCMSC treated traumatic rats exhibited significantly increased numbers of Brdu immunoreactive cells in their traumatic core compared with other labeled group (*P*< 0.01).

Group	PBS	Brdu	G-CSF	hUCMSC	G-CSF+hUCMSC
Cell Count	0.0	11.10±2.07	31.46±7.28	33.20±5.76	42.23±6.46

## Discussion

In the present study we proved that rats hUCMSC injected intravenously survived until 6 weeks after treatment, and that hUCMSC treatment improves functional recovery until 6 weeks after treatment. Our data shows that functional benefit induced by hUCMSC treatment was visible at 1 week after treatment and continued until the end of the trial (6 weeks). This benfit of hUCMSC treatment proved the effectiveness of hUCMSC in improving functional recovery. Many inquiries have suggested that the “injured” brain might inhale stem cells or those stem cells might “home in” on the injured brain (26,27). Stumm *et al* proved that focal cerebral ischemia express SDF-1 (stromal derived factor-1) (28). SDF-1 is a CXC chemokine steady produced by bone marrow stromal cells and is a potent chemo-attractant for stem cells. In a study (29) a lesion-induced up regulation of endothelial SDF-1 (RT-PCR results) and the appearance of increased bis-benzimide-labeled CXC receptor 4-expressing cells in the ischemic hemisphere proved that cerebro-endothelial SDF-1 is a chemo-attractant for hUCMSC. Moreover, bis-benzimide-labeled stem cells, co-stained with a definite marker for doublecortin (30) and transplanted in the striatum, were shown to migrate into the penumbra of the ischemic cortex 1 month after transplantation. This result indicates that, after traumatic brain injury, hUCMSC were directed toward sites of brain injury, where they causes brain repair and functional recovery. Our data also show that traumatic brain tissue induced migration and homing of hUCMSC, which in turn suggests that traumatic-induced chemotaxis may target hUCMSC to damaged tissues by attracting hUCMSC to the traumatic region, an SDF-1/CXCR4 interaction may be directly involved in vascular remodeling, angiogenesis and neurogenesis, thereby alleviating traumatic symptoms. It has been proved that mRNA rate of neurotrophic factors comprising SDF-1, BDNF (brain derived neurotrophic factor) and GDNF (glial derived neurotrophic factor) intensified markedly 7 days after transplantation in hUCMSC-treated rats’ brains in comparision with the control group (29). Hence, we guess that the grafted hUCMSC and their migration into the injured tissue may induce a neurotrophic factor secretion or interact with the host brain resulting in hUCMSC and parenchyma cells producing neurotrophic factors which may take part in functional recovery of the traumatic brain. HUCMSC may induced glial cells to secrete neurotrophins (BDNF and NGF (neural growth factor)) (30). G-CSF mobilizes bone marrow cells that are a compound of hematopoietic progenitor cells (mainly CD34+),(31). Besides to the mobilization of hematopoietic progenitor cells, G-CSF itself might have so many ways to repair neuronal injuries: G-CSF receptors are recognized on neurons and glia, so G-CSF may have neuroprotective effect on these cells (32). Hence, G-CSF may causes neuroprotection after TBI by prohibitory excitotoxicity and stimulating the transcription of neuroprotective genes (32). G-CSF (filgrastim) has been applied after neuronal damage to decrease the peril of sepsis in patients with traumatic brain injury (33). It was proved that the number of white blood cells in the peripheral blood enhances after G-CSF treatment (31). Hence, G-CSF may not only have advantage on brain tissue, but can likewise tend to increase the amount of systemic neutrophils. This neutrophilia can then have a side effect on the expansion of a traumatic damage tissue as portion of the inflammatory reactions in the injured tissue. This opinion is in correspond with Fukumoto’s discovery, in which he and his colleagues proved that G-CSF application causes aggregation of inflammatory cells that stimulate cerebral and myocardial infarctions (34). In against, Taguchi (35) found that CD34^+^ cells induce a positive role in neuroregeneration by stimulating neovascularization in the ischemic area of the mouse brain, providing growth factors or cytokines and providing a pleasure milieu for neurogenesis. In the brain the stimulus of CD34+ cells mobilized by G-CSF may have the similar mechanisms. Many researchers declare that a combination of tactics may be desired to prohibit tissue lesion and increase regeneration and reconnection. For instance Karim *et al* used combination of a agent for reducing inhibitory effect of the scar tissue (chondroitinase ABC), and schwann cell accompany with olfactory-ensheathing glia grafts (38). In our study we recognized that the effect of G-CSF and hUCMSC usage had minority effectiveness than combine therapy. Our data proved that all two groups’ hUCMSC and G-CSF medication were almost identical influence on enhancing functional result with no marked difference between them. Although the number of cells discovered in injured brain was markedly additive with hUCMSC than with G-CSF. HUCMSc transplantation does not cause soon or late xenograft rejection, this may be due to poor immunogenicity of hUCMSC (36,37). Therapeutic advantage of G-CSF and hUCMSC in combination may induce an additive profit to augment angiogenesis and neurogenesis. The augmented angiogenesis and neurogenesis stimulated by the combination of hUCMSC and G-CSF may be imputed to the supplementary and plus stimulus of growth factor by them. Biological transplantation using hUCMSC or combine therapy with G-CSF as implement of stimulating neural repair to treat TBI and neural injury is anongoing advanced therapy. Nevertheless, before these laboratory finding can be applied into clinical therapeutic use, queries for instance proper dose and long term stimulus both toxic and advantageous must to be reconsidered.

## Conclusion

In brief, our data suggest that G-CSF interaction with hUCMSC may be promotes angiogenesis and neurogenesis after TBI that may benefit neurological functional recovery. Nevertheless, other questions, for example the molecular and genetic origin of this neural recovery caused by hUCMSC need more investigations. This therapeutic improvement of combination treatment may be imputed to the increased plasticity. These data offer that pharmacological therapy may intensify cellular therapy and produce therapeutic advantage after TBI. 
